# ORACLE reveals a bright future to fight bacteria

**DOI:** 10.7554/eLife.68277

**Published:** 2021-04-15

**Authors:** Willow Coyote-Maestas, James S Fraser

**Affiliations:** 1 Department of Bioengineering and Therapeutic Sciences, UCSFSan FranciscoUnited States; 2 Quantitative Biosciences Institute, UCSFSan FranciscoUnited States

**Keywords:** bacteriophage, phage, virus engineering, deep mutational scanning, synthetic biology, tail fiber, Virus

## Abstract

A new way to alter the genome of bacteriophages helps produce large libraries of variants, allowing these bacteria-killing viruses to be designed to target species harmful to human health.

**Related research article** Huss P, Meger A, Leander M, Nishikawa KK, Raman S. 2021. Mapping the functional landscape of the receptor binding domain of T7 bacteriophage by deep mutational scanning. *eLife* **10**:e63775. doi: 10.7554/eLife.63775

Antibiotics are usually quite effective at killing bacteria that cause disease, but they often end up eliminating huge swaths of microorganisms beneficial to health . Limiting this collateral damage by solely targeting pathogenic bacteria remains challenging, as only slight differences separate harmful and beneficial bacterial species.

An alternative treatment to chemical antibiotics could be to harness viruses called bacteriophages (or phages), which have evolved to recognize and prey on highly specific strains of bacteria (Abedon et al., 2011). Yet engineering phages to target harmful bacterial species requires scalable genetic tools that can precisely alter the genomes of these viruses. Now, in eLife, Srivatsan Raman and colleagues at University of Wisconsin-Madison – including Phil Huss as first author – report a new method that can create thousands of mutations in a host-specifying phage protein, showing how these changes alter which bacteria the virus can target (Huss et al., 2021).

Before this study, most protein engineering tools that ‘tweaked’ the genome of a bacteriophage to create desirable traits relied on either rational or directed evolution methods (Favor et al., 2020; Yehl et al., 2019). In rational approaches, the detailed knowledge of the three-dimensional structure of a protein – and its associated function – helps to guide mutations that will lead to desired properties. Directed evolution approaches, in contrast, bypass the need for such detailed understanding as they involve making random mutations which are then filtered for beneficial effects through multiple rounds of selection. However, these methods tend to only sample a small proportion of possible mutations.

Another approach to identify beneficial genetic variation is deep mutational scanning (DMS for short), where every single amino acid in a protein is changed to all other amino acids to see how it affects the function of a protein. This creates both a mechanistic structure-function understanding of the protein, and a starting point for protein engineering. DMS guided-methods are becoming widely used in protein engineering to develop better antibodies, enzymes and virus-based gene delivery systems (Adams et al., 2016; Ogden et al., 2019; Romero et al., 2015). Until the work by Huss et al., however, DMS approaches in bacteriophages were limited by a lack of methods to build sizeable and comprehensive mutational libraries in bacteriophage genomes.

To address this problem, the team developed the ORACLE method (short for Optimized Recombination Accumulation and Library Expression), a new phage genome engineering approach which creates large and unbiased mutational libraries of a phage gene. ORACLE can produce complete libraries of thousands of mutations, enabling DMS-based approaches in phages. The approach was used to create a library of mutant bacteriophages that each carried a single amino acid mutation in the tip of their receptor binding protein (known as RBD), the structure the viruses use to recognize their target species.

Massively parallel DNA sequencing was then applied to assess which mutant bacteriophages multiplied after being exposed to various strains of bacteria, revealing how each amino acid mutation in the RBD tip contributed to host specificity. In particular, changes in amino acids exposed to the surface and in contact with the host gave rise to phage variants highly specific to different bacterial strains.

Additional experiments further showcased this remarkable specificity, and the power of ORACLE. In particular, the phage library obtained through the DMS approach was tested on bacterial strains that carried on their surface slightly modified versions of the sugar molecules (or lipopolysaccharide) recognized by the viruses. This allowed Huss et al. to find phage variants whose ability to infect their prey depended on the type of lipopolysaccharides present on the bacteria. Finally, the team demonstrated the potential therapeutic utility of this approach, selecting for and characterizing RBD tip mutations that made phages target a strain of bacteria which causes urinary tract infections. This work by Huss et al. demonstrates how DMS-based approaches can help to understand the basic principles underlying bacteriophage specificity, and dramatically improve their ability to prey on pathogenic bacteria.

New functions often emerge when multiple genetic changes accumulate and interact, creating larger (or smaller) effects on protein function than would be expected from the sum of individual mutations. DMS experiments alone are ill suited to examine those interactions, as it is impractical to make all double, triple, and other higher-order mutations. However, machine-learning models trained on DMS datasets can help to explore these higher order interactions and to predict useful multi-mutational variants. This approach was applied, for instance, to help turn a small virus into a tool to deliver genes of interest to specific mammalian cells (Ogden et al., 2019). Such DMS-trained models could be employed for bacteriophages in order to expand or specify the host range beyond what is possible with single mutations alone.

A lack of genetic tools and the sheer number of bacteriophage species have turned these viruses into the understudied dark matter of the viral universe. Ongoing ‘metagenomic’ studies that sample the genetic material of phages in the natural world are revealing many strains whose biology is unknown, and which carry massively diverse sequences (Jordan et al., 2014). ORACLE is unlocking new ways to characterize bacteriophages, paving the way to one day harness these sequences to understand and engineer the microbial world within and around us.
